# Supplementary material for: Splice-Junction-Based Mapping of Alternative Isoforms in the Human Proteome
Source: Cell Rep. Author manuscript; Available in PMC 2020 Jan 15. (PMC6961840; doi:10.1016/j.celrep.2019.11.026)
Supplement: 2 [file NIHMS1546469-supplement-2.zip › DF1/PXD010154/Thyroid/Thyroid_45_AFMID_QQTLCQGEWK.pdf]

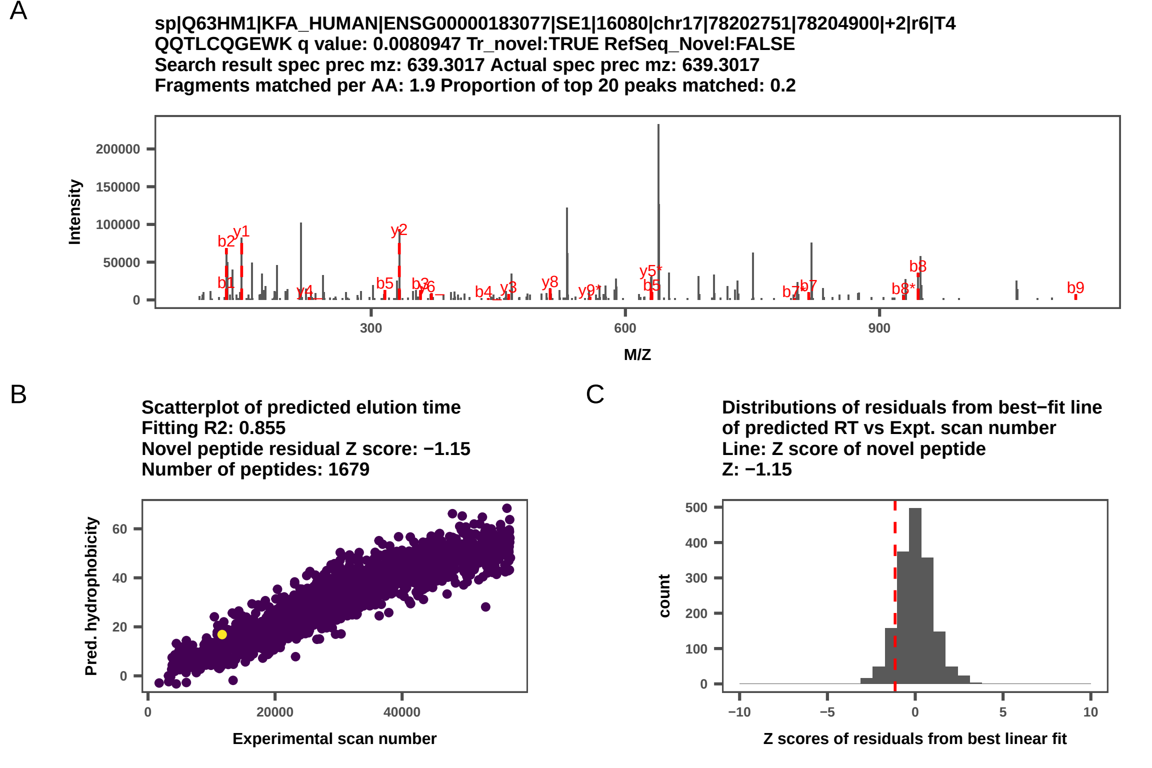
A
sp|Q63HM1|KFA_HUMAN|ENSG00000183077|SE1|16080|chr17|78202751|78204900|+2|r6|T4
QQTLCQGEWK q value: 0.0080947 Tr_novel:TRUE RefSeq_Novel:FALSE
Search result spec prec mz: 639.3017 Actual spec prec mz: 639.3017
Fragments matched per AA: 1.9 Proportion of top 20 peaks matched: 0.2
Intensity
200000
150000
100000
50000
0
b2
y1
b1
y4
y2
b5
b3
y6
b4
y3
y8
y9*
y5*
b5
b7*
b7
b8
b8*
b9
300
600
900
M/Z
B
Scatterplot of predicted elution time
Fitting R2: 0.855
Novel peptide residual Z score: −1.15
Number of peptides: 1679
Pred. hydrophobicity
60
40
20
0
0
20000
40000
Experimental scan number
C
Distributions of residuals from best−fit line
of predicted RT vs Expt. scan number
Line: Z score of novel peptide
Z: −1.15
count
500
400
300
200
100
0
−10
−5
0
5
10
Z scores of residuals from best linear fit
